# Mesenchymal stem cell therapy in perinatal arterial ischemic stroke: systematic review of preclinical studies

**DOI:** 10.1038/s41390-022-02208-3

**Published:** 2022-07-29

**Authors:** Verena Lehnerer, Anna Roidl, Olga Romantsik, Raphael Guzman, Sven Wellmann, Matteo Bruschettini

**Affiliations:** 1 Department of Neonatology, University Children’s Hospital Regensburg (KUNO) at the Hospital St. Hedwig of the Order of St. John, University of Regensburg, Regensburg, Germany; 2 Department of Clinical Sciences Lund, Paediatrics, Lund University, Skåne University Hospital, Lund, Sweden; 3 Faculty of Medicine, University of Basel, 4056 Basel, Switzerland; 4 Department of Neurosurgery, University Hospital Basel, 4031 Basel, Switzerland

## Abstract

**Background:**

Perinatal arterial ischemic stroke (PAIS) is a neurologic disorder leading to long-term complications. Mesenchymal stem cells (MSCs) have emerged as a novel therapeutic agent. This systematic review aims to determine the effects of stem cell-based interventions for the treatment of PAIS in preclinical studies.

**Methods:**

We included all controlled studies on MSCs in neonatal animals with PAIS. Functional outcome was the primary outcome. The literature search was performed in February 2021.

**Results:**

In the 20 included studies, MSCs were most frequently delivered via intracerebral injection (*n* = 9), 3 days after the induction of PAIS (*n* = 8), at a dose ranging from 5 × 10^4^ to 5 × 10^6^ cells. The meta-analysis showed an improvement on the cylinder rearing test (MD: −10.62; 95% CI: −14.38 to −6.86) and on the water maze test (MD: 1.31 MD; 95% CI: 0.80 to 1.81) in animals treated with MSCs compared to the control group animals.

**Conclusion:**

MSCs appear to improve sensorimotor and cognitive performance in PAIS-injured animals; however, the certainty of the evidence is low. Registration of the protocol of preclinical studies, appropriate sample size calculation, rigorous randomization, and reporting of the data on animal sex and survival are warranted.

PROSPERO registration number: CRD42021239642.

**Impact:**

- This is the first systematic review and meta-analysis of preclinical studies investigating the effects of MSCs in an experimental model of PAIS.
- MSCs appear to improve sensorimotor and cognitive performance in PAIS-injured neonatal animals.
- The certainty of the evidence is low due to high or unclear risk of bias in most domains.

## Introduction

Ischemic perinatal stroke has been defined as a focal disruption of cerebral blood flow that takes place between 20 weeks of gestation and 28 postnatal days.^1^ The incidence of perinatal arterial ischemic stroke (PAIS) from population-based data ranges between 10 and 29 per 100,000 live births.^2–4^ Several independent risk factors such as male sex, chorioamnionitis, multiple births, preterm birth, and small for gestational birth have recently been implicated in the etiopathogenesis of PAIS.^5,6^ Frequently, neonates with PAIS present with seizures within the first days after birth and may be accompanied by (asymmetric) hypotonia, lethargy, and apnea.^7^ Overall, outcomes from perinatal stroke are poor, with most patients developing lifelong neurological disabilities.^7^ In 50–75% of infants, PAIS leads to abnormal motor and neurodevelopmental outcomes, including cerebral palsy, cognitive dysfunction, behavioral disorders, and epilepsy.^7^ Current treatment options for PAIS consist only of supportive care, such as controlling hypoglycemic and seizures. As these treatments offer only symptomatic care and no cure, additional therapeutic strategies for PAIS are urgently needed.

Mesenchymal stem cells (MSCs) have emerged as novel therapeutic agents with promising results in experimental studies of newborns. The therapeutic potential of MSCs in brain injuries has mainly been attributed to their immunomodulatory and regenerative potential.^8^ Several preclinical studies provide evidence for the use of MSC-based therapy in the neonatal period. Most intensively the condition of bronchopulmonary dysplasia (BPD) in the neonatal lung has been studied. Two recently published systematic reviews on preclinical trials showed a significant therapeutic benefit of MSCs therapy on several outcome measures and suggest that MSCs are the most effective therapy for BPD.^9,10^ Similarly, for neonatal brain pathologies including the condition of hypoxic-ischemic encephalopathy (HIE)^11,12^ and intraventricular hemorrhage (IVH),^13^ MSCs were reported to have positive effects on neurobehavioral outcomes, repairing brain tissue and attenuating brain damage. There is also a growing number of preclinical studies investigating the potential therapeutic role of MSCs in experimental PAIS.^14^ To date, there has been no systematic review and/or meta-analysis on the therapeutic potential of stem cells in experimental PAIS.

This systematic review and meta-analysis aimed to evaluate and summarize the available evidence on the therapeutic potential and safety of MSCs in neonatal animal models of PAIS.

## Methods

Our methods for systematically reviewing the preclinical studies are based on the tools and guidelines offered by SYRCLE. The protocol (CRD42021239642) was registered on PROSPERO before starting the review. We used the Preferred Reporting Items for Systematic Reviews and Meta-Analyses (PRISMA) checklist for the manuscript.^15^

### Search strategy

We conducted a comprehensive search including MEDLINE via PubMed (818 records), Embase (532 records), and Web of Science (2028 records) on February 19, 2021. The search strategy involved the following search components: mesenchymal stem cells, stroke, and animals (for the full search strategies for each database, see S1). We used no language or publication date restriction. Duplicates were automatically indicated by EndNote and removed.

### Inclusion criteria

We included preclinical, randomized, and non-randomized controlled studies of neonatal animal models mimicking PAIS. “Neonatal” was defined as the first 10 days since birth, as this time interval has been referred to as the neonatal period, at least in rodents.^16^ We included all studies that evaluated the therapeutic potential and safety of MSCs. MSCs were defined using the minimal criteria set out in the International Society for Cellular Therapy (ISCT) consensus statement.^17^ Non-interventional studies, studies without controls, and non-neonatal models of PAIS were excluded.

### Study selection

Two authors (V.L. and A.R.) independently screened titles and abstracts for inclusion using the Software Covidence. For the potentially relevant articles, the full text was retrieved, and eligibility was assessed according to our inclusion criteria. Disagreements about inclusion were resolved by discussion and consensus among a third author (M.B.).

### Data extraction

Two reviewers (A.R. and O.R.) extracted the data using a predetermined data extraction sheet. A third reviewer checked the data for accuracy (V.L.). Data were extracted for study characteristics (authors, year of publication, and study location), study design (sample size for intervention, control, and sham groups), intervention characteristics (timing, dose, and mode of stem cell administration), and outcome measures (primary and secondary outcomes). Dichotomous and continuous data provided in numbers were extracted directly. As most of the data were available in figures and not in numerical form, we used a validated graphical digitizer (WebPlotDigitizer),^18^ an open-source program that can work with a variety of plot types and images. First, the images of the figures for the relevant outcome from all included studies were saved as screenshots, then these images were uploaded to the application. The first step of the analysis consisted of defining the type of graph analyzed, which was typically a two-dimensional bar plot, and calibrating the axis by assigning four points of known values on the axis. Then, the data points were extracted.

### Outcomes

Our primary outcomes were any functional outcome measure that was defined as a quantified measure across any of the WHO-ICF domains of impairment, activity (disability), or participation (handicap);^19^ mortality during the study; and any rate of adverse events and harms.

Our secondary outcomes were inflammation markers for the brain, lesion size as measured by neuroimaging or by immunohistochemistry (IHC), and outcomes and/or markers for neurogenesis, apoptosis, and neuronal development.

### Assessment of risk of bias

We used SYRCLE’s risk of bias tool^20^ for animal studies to assess the risk of bias in the included studies. Two reviewers (A.R. and V.L.) independently evaluated the studies, any disagreements were solved through discussion and, if necessary, by consulting a third review author (M.B.). The following domains were assessed as low risk of bias, high risk of bias, or unknown risk of bias: selection bias due to sequence generation; baseline characteristics or inadequate allocation concealment; performance bias due to inadequate randomization housing or blinding; detection bias due to inadequate randomization of outcome assessment or blinding; attrition bias due to incomplete outcome data; reporting bias due to selective outcome reporting; and other sources of bias. We adapted the GRADE methodology to assess the certainty of the evidence for the main outcomes.

### Data analysis

Data were analyzed with the Cochrane Software RevMan 5.4.^21^ We calculated standard deviations from standard errors and *n* values. We used mean difference (MD) for the continuous outcomes. Due to anticipated heterogeneity, summary statistics were calculated with a random-effects model. We assessed statistical heterogeneity with the *I*^2^ statistic with 95% confidence intervals and data were visualized using forest plots. Statistical heterogeneity was assessed as very low (0–25%), low (25–50%), moderate (50–75%), and high (>75%) using the *I*‐statistic.

### Subgroup analyses

We planned to perform the following subgroup analyses: sex (male and female); dose of MSCs, i.e., high dose and low dose; route of administration: intravenous, intraventricular, intranasal; number of administrations: 1, 2–5, >5; and timing of administration: early (postnatal day 0–2), late (postnatal day 3–9), very late (postnatal day 10).

## Results

**Fig. 1:**
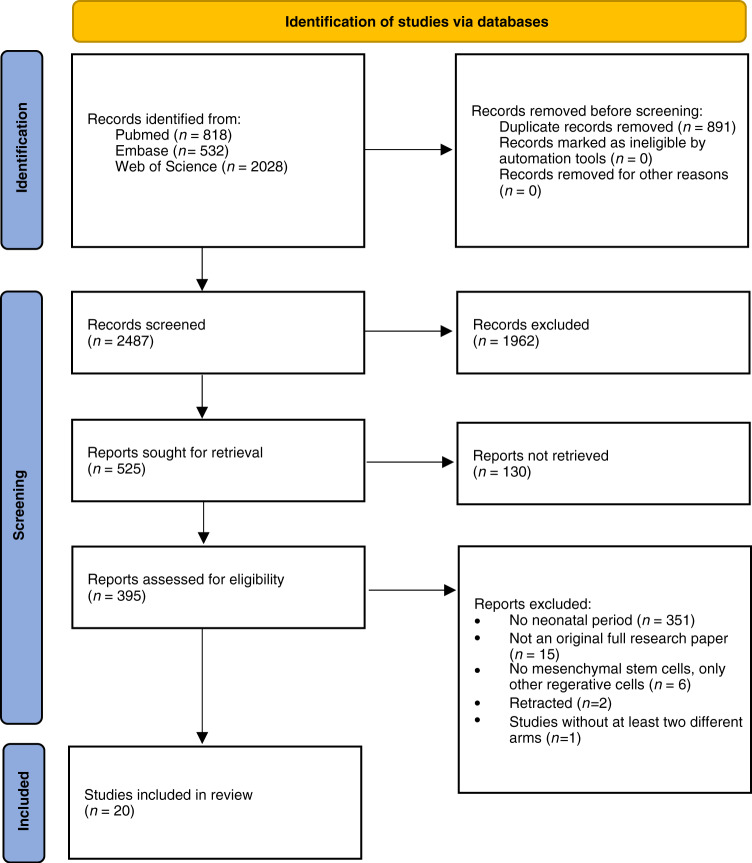
PRISMA flowchart.

Our search for animal studies returned a total of 3378 records and 891 duplicates were removed, resulting in 2487 studies for screening. Figure 1 shows the PRISMA diagram of the comprehensive search and the reasons for excluding studies. Following screening titles and abstracts, 525 studies were selected and screened for full text. For 130 studies no full text was available; of the remaining studies, 395 studies were excluded mainly because animals were not in the neonatal period (*n* = 351). Further reasons were that the studies were not an original full research paper (*n* = 15), only other regenerative cells than MSCs were used (*n* = 6), two studies have been retracted and one study had no different study arms.

### Study characteristics and study population

The 20 studies included in this review were published between the years 2010 and 2021. The characteristics of included studies are summarized in Table 1. Among the included articles, seven were from China,^22–28^ five from the Netherlands,^29–33^ three from Korea^34–36^ and the USA,^37–39^ and two from Japan.^40,41^ Rodents exposed to PAIS were the only animal model. The rat being the most used species (*n* = 14)^22–28,33,35–39,41^ followed by mouse (*n* = 6).^29–32,34,40^ Regarding the sex of the animals, half of the studies did not report this information (*n* = 10), three studies only used male pups^35,36,39^ and seven studies^24,31,32,34,37,38,41^ used pups of both sexes. None of the included studies investigated the effect of MSCs in females alone, nor did comparative analysis. All studies induced PAIS within postnatal day 10, most of the studies on postnatal day 7 (*n* = 11).^22–28,34,36,39,41^ In all, 70% (*n* = 14) of all studies used a combination of occlusion either of carotid or internal carotid artery followed by hypoxia. Among these trials, ten^22–26,28,34,36,40,41^ used a mixture of 8% O_2_ for hypoxia, while the four trials^29–32^ induced hypoxia with 10% O_2_. The remaining studies (*n* = 5)^33,35,37–39^ used either carotid artery, middle cerebral artery, or internal carotid artery occlusion. One study^24^ differed from all the others by using reperfusion after 30 min.

**Table 1:** Characteristics of included studies.

Author (year); Country	Animal characteristics		PAIS model	Intervention characteristics			Control group
	Species, Strain; Sex; Age	Age		MSCS source; Type	MSCS dose; Frequency	Day of administration; Mode	
Cho et al. (2016);^34^ Korea	Mouse; CD-1 (ICR); 0	7 PND	Ligation rCCA; hypoxia (8% O_2_, 90 min)	NR	1 × 10^5^ cells; single	PND 42; intrastriatal	PBS; PBS-EE; MSC-EE
Ding et al. (2015);^22^ China	Rat; Wistar; NR	7 PND	Double ligation + cut lCCA; 2 h recovery; hypoxia (8% O_2_, 2.5 h)	Placenta-derived MSCs	5 × 10^5^ cell/μL; single	PND 11; intracerebral	HIBD; HIBD + fibroblasts; Sham
Ding et al. (2017);^23^ China	Rat; Wistar; NR	7 PND	Cut LCCA; hypoxia (8% O_2_, 2.5 h)	Placenta-derived MSCs	5 × 10^4^ cells/10 µL; single	PND 9; intracerebral	HIBD; HIBD + fibroblast group; Sham
Kim et al. (2012);^35^ Korea	Rat; Sprague–Dawley; 1	10 PND	Occlusion with silicone rubber; rMCA	MSCs from human umbilical cord	1 × 10^5^ cells; single	PND 10; intraventricular	PBS; Sham
Larpthaveesarp et al. (2021);^37^ USA	Rat; Sprague–Dawley; 0	10 PND	Dissection + 3 h occlusion with silicon-coated nylon filament; rMCA	Adipose-tissue-derived MSCs	3 × 10^6^ cells; single	PND 13; PND 17; intranasal	Sham; MCAO + media; MCCO + 3d MSC/EPO; MCAP + 3d EPO3; MCAO + 7d MSC/EPO; MCAP + 7d EPO3
Lee et al. (2010);^36^ Korea	Rat; Sprague–Dawley; 1	7 PND	Double ligation + cut lCCA; 2 h recovery; hypoxia (8% O_2_, 3.5 h)	Human bone marrow mesenchymal stem cells	1 × 10^6^ cells; single	PND 10; intracardial	Sham + buffer; Sham-MSC; Hi-buffer
Li et al. (2014);^24^ China	Rat; Sprague–Dawley; 0	7 PND	30 min ligation with reperfusion (4.0 suture); lCCA; NR; hypoxia (8% O_2_, 2.5 h)	Rats bone marrow mesenchymal stem cells	1 × 10^6^ cells; single	PND 8; intraperitoneal	Sham; PBS; Photon + MSC
Ohshima et al. (2015);^40^ Japan	Mouse; CB17; NR	8 PND	Permanent occlusion; lCCA; 1 h recovery; hypoxia (8% O_2_, 30 min)	Rats bone marrow mesenchymal stem cells	1 × 10^6^ cells; single	PND 10; intravenous; intraperitoneal	MNC-intravenous; MNC-intraperitoneal
Sakai et al. (2018);^41^ Japan	Rat; Sprague–Dawley; 0	7 PND	double ligation (6.0 silk suture) and section lCCA; 1 h recovery; hypoxia (8% O_2_, 2 h)	Rats bone marrow mesenchymal stem cells	1 × 10^6^ cells; single	PND 10; intravenous	Sham; Vehicle
van Velthoven et al. (2017);^38^ USA	Rat; Sprague-Dawley; 0	10 PND	1.5 h ligation (6.0 dermalon filament); ICA ; no hypoxia	Rat Sprague–Dawley bone marrow mesenchymal stem cells (GIBKO®)	1 × 10^6^ cells; single	PND 13; intranasal	Sham-Vehicle; Sham-MSC; Vehicle
van Velthoven et al. (2013);^33^ Netherlands	Rat; Sprague-Dawley; NR	10 PND	1.5 h double ligation (6–0 silk suture) and dissection of ICA	Rat Sprague–Dawley bone marrow mesenchymal stem cells (GIBKO®)	1 × 10^6^ cells; single	PND 13; intranasal	Sham-Vehicle; Sham-MSC; Vehicle; MSC-BDNF
van Velthoven et al. (2010);^29^ Netherlands	Mouse; C57Bl/6; NR	9 PND	Occlusion; right carotid artery; hypoxia (10% O_2_, 45 min)	Bone marrow from femur and tibia of 6- to 8-week-old C57BL/6-Tg (UBC-GFP) 30 Sch/J mice	1 × 10^5^ in 2 µL PBS; single and repeated	PND 12; PND 17; intracerebral	Sham; Vehicle
van Velthoven et al. (2010);^31^ Netherlands	Mouse; C57Bl/6J; 0	9 PND	Permanent occlusion; right carotid artery; hypoxia (10% O_2_, 45 min)	Bone marrow from femur and tibia of 6- to 8-week-old C57BL/6-Tg (UBC-GFP) 30 Sch/J mice	5 × 10^5^ in 12 µL PBS	PND 17; intranasal	Vehicle
van Velthoven et al. (2010);^32^ Netherlands	Mouse; C57Bl/6; 0	9 PND	Occlusion; right carotid artery; hypoxia (10% O_2_, 45 min)	Bone marrow from femur and tibia of 6- to 8-week-old C57BL/6-Tg (UBC-GFP) 30 Sch/J mice	1 × 10^5^ in 2 µL PBS	PND 12; intracerebral	Sham; Vehicle
van Velthoven et al. (2012);^30^ Netherlands	Mouse; C57Bl/6J; NR	9 PND	Occlusion; right carotid artery; hypoxia (10% O_2_, 45 min)	Bone marrow from femur and tibia of 6- to 8-week-old C57BL/6-Tg (UBC-GFP) 30 Sch/J mice	1 × 10^5^ in 2 µL PBS; repeated	PND 10; PND 17; intracerebral	Sham; Vehicle
Wei et al. (2015);^39^ USA	Rat; Wistar; 1	7 PND	Permanent ligation (10 silk sutures); distal branches MCA; cauterization rCCA	Bone marrow mesenchymal stem cells from Wistar rats – hypoxia preconditioned	1 × 10^6^ cells in 100 µL; repeated	PND 7; PND 10; intranasal	Shame; Saline
Xia et al. (2010);^25^ China	Rat; Sprague–Dawley; NR	7 PND	Permanent ligation (6–0 surgical silk) lCCA; 3 h recovery; hypoxia (8% O_2_, 2.5 h)	Human umbilical cord blood mesenchymal stem cells	5 × 10^4^ µL; single	PND 10; intrecerebroparenchymal	Culture medium
Yang et al. (2020);^28^ China	Rat; Sprague-Dawley; NR	7 PND	Permanent ligation (6–0 surgical silk) lCCA; 3 h recovery; hypoxia (8% O_2_, 2.5 h)	NR	2 × 10^5^ cells; single	PND 7; intracerebroventricular	Sham; PBS; siIL-6 MSCs; GFP MSCs
Zhang et al. (2014);^26^ China	Rat; Sprague-Dawley; NR	7 PND	Ligation (6–0 surgical silk rCCA; 2–3 h recovery; hypoxia (8% O_2_, 2 h)	Human umbilical cord mesenchymal stem cells (Wharton’s jelly)	5 × 10^5^ cells; single; 5 × 10^6^ cells; single	PND 8; PND 10; intravenous; intraperitoneal; different groups	Sham-Vehicle; HIE-vehicle 24 h; HIE-DFB
Zhou et al (2015);^27^ China	Rat; Sprague-Dawley; NR	7 PND	According to Rice,^21^ no further description	Human umbilical cord mesenchymal stem cells	2 × 10^5^ cells; single	PND 12; intracerebroventricular	PBS; Sham

0 = both sexes, 1 = male, 2 = female, *NR* not reported, *PND* postnatal day, *rCCA* right common carotid artery, *lCCA* left common carotid artery, *MCA* middle cerebral artery, *MSC* mesenchymal stem cell, *MNC* mononuclear cell, *PBS* phosphate-buffered saline, *HIE* hypoxic-ischemic encephalopathy, *HIBD* hypoxic-ischemic brain damage, *EE* enriched environment, *EPO* erythropoietin, *DFB* dermal fibroblasts, *GFP* green fluorescent protein, *BDNF* brain-derived neurotrophic factor.

### MSCs characteristics and application

As shown in Table 1, intracerebral injection of MSCs was the most common route of delivery (*n* = 10),^22,23,25,27–30,32,34,35^ followed by intranasal inhalation (*n* = 5),^31,33,37–39^ intravenous infusion (*n* = 3),^26,40,41^ intraperitoneal (*n* = 3),^24,26,40^ and intracardial (*n* = 1)^36^ injection. Of these, two studies compared two routes of application (intravenous vs. intraperitoneal).^26,40^ Almost all studies (*n* = 17) investigated only a single dose of MSCs, apart from three studies^29,30,39^ that studied a repeated administration of MSCs, after either 3, 5, or 7 days following the first application. In eight studies,^25,29,31,33,36–38,41^ the first administration of MSCs was done 3 days after the induction of PAIS, six studies applied the MSCs therapy on the same day (*n* = 3),^28,35,39^ or 1 day (*n* = 3)^24,26,30^ after the induction of PAIS. In two studies,^23,40^ MSCs therapy was administered 2 days after the induction of PAIS. The remaining studies (*n* = 4) used a later application of MSCs at 4,^22^ 5,^27^ 8,^32^ and 35^34^ days following PAIS. The doses ranged from 5 × 10^4^ cells to a maximum of 5 × 10^6^ cells, while most often 1 × 10^6^ cells were used (*n* = 7).^24,33,36,38–41^ Bone marrow was the most common source of MSCs (*n* = 11).^24,29–33,36,38,40,41^ Further MSCs were derived from human umbilical cord blood (*n* = 1),^25^ from umbilical cord (*n* = 2),^27,35^ placenta (*n* = 2),^22,23^ Wharton’s jelly (*n* = 1),^26^ and adipose tissue (*n* = 1).^37^ Two studies did not specify the source of MSCs.^28,34^ A total of 39% (*n* = 7) studies performed xenogeneic transplant, while 56% (*n* = 10) performed allogeneic transplantation. For three studies there was no information available.

All studies except one^40^ used the PAIS model without additional injection, with the administration of phosphate-buffered saline, saline, or vehicle as a control group. Sixteen studies^22–24,26–31,33,35–39^ also compared the MSCs groups with healthy, non-, sham-operated animals.

Table 2 summarizes the characterization of the cells used in the animal experiments. Using the ISCT criteria, only four studies reported information for all categories.^22,23,25,27^ Plastic adherence was reported in 90% (18 studies). Positive and negative markers specific to MSCs were confirmed in 80% (*n* = 16) of studies. Six of these studies^29–33,38^ that reported negative markers identified them simply as myeloid and hematopoietic cell lineage-specific antigens, rather than naming specific markers. The ability to differentiate into various cell lineages was reported in seven studies.^22,23,25,27,31,35,37^

**Table 2:** MSCs criteria reported by included studies.

Author (Year)	Does the study report MSCS criteria?	Were MSCs purchased or supplied?	Plastic adherence?	Positive markers	Negative markers	Differentiation capability	Cell expansion media	Passage number
Cho et al. (2016)^34^	No (only plastic adherence)	Not reported	Yes	Not reported	Not reported	Not reported	DMEM-low glucose, 10% FBS, penicillin, streptomycin	Not reported
Ding et al. (2015)^22^	Yes	No	Yes	CD 29, CD 44, CD 90, CD 105	CD54	Osteoblasts, chondrocytes, adipocytes	Minimum essential medium-α, 10% FBS, penicillin, streptomycin	4–5
Ding et al. (2017)^23^	Yes	No	Yes	CD 29, CD 44, CD 90, CD 105	CD 45	Osteoblasts, chondrocytes, adipocytes	Minimum essential medium-α, 10% FBS, penicillin, streptomycin	4–5
Kim et al. (2012)^35^	Yes	No	Yes	Oct-4, SSEA-4, HLA-AB, CD 73, CD 105	HLA-DR, CD 14, CD 34, CD 45	Respiratory epithelium, osteoblasts, chondrocytes, adipocytes	Not reported	5
Larpthaveesarp et al. (2021)^37^	Yes	Yes (Creative Bioarray, Shirley, NY)	Not reported	CD 29, CD 44, CD 90, and CD 105	CD11b, CD 34, CD 45	Osteogenic, chondrogenic, adipogenic lineages	Not reported	Not reported
Lee et al. (2010)^36^	Yes	No	Yes	CD 73, CD 105	CD 14, CD 34, CD 45	Not reported	10% DMEM-low glucose, 10% FBS, 1% antibiotic-antimycotic solution	Within 5
Li et al. (2014)^24^	No (only plastic adherence)	No	Yes	Not reported	Not reported	Not reported	DMEM/F12, 10% FBS	4–6
Ohshima et al. (2015)^40^	No (only plastic adherence)	No	Yes	Not reported	Not reported	Not reported	α-MEM, 10% FBS, penicillin, streptomycin	4–5
Sakai et al. (2018)^41^	Yes	No	Yes	CD 73, CD 90	CD 45, CD 106	Not reported	DMEM, 10% heat-inactivated FBS, L-glutamine, penicillin, streptomycin	3
van Velthoven et al. (2017)^38^	Yes	GIBCO	Not reported	CD 29, CD 44, CD 90, CD 106	Myeloid and hematopoietic cell lineage-specific antigens	Not reported	Not reported	Not reported
van Velthoven et al. (2013)^33^	Yes	GIBCO	Yes	CD 29, CD 44, CD 90, CD 106	Myeloid and hematopoietic cell lineage-specific antigens	Not reported	DMEM	Not reported
van Velthoven et al. (2010)^29^	Yes	The Jackson Laboratory	Yes	Sca-1, MHC-I, CD 29, CD 44, CD 90	Myeloid and hematopoietic cell lineage-specific antigens	Not reported	DMEM, 15% FBS	Not reported
van Velthoven et al. (2010)^31^	Yes	The Jackson Laboratories	Yes	Sca-1, MHC-I, CD 29, CD 44, CD 90	Myeloid and hematopoietic cell lineage-specific antigens	Not reported	DMEM, 15% FBS	Not reported
van Velthoven et al. (2010)^32^	Yes	The Jackson Laboratory	Yes	Sca-1, MHC-I, CD 29, CD 44, CD 90	Myeloid and hematopoietic cell lineage-specific antigens	Adipocytes, osteocytes, chondrocytes	DMEM, 15% FBS	Not reported
van Velthoven et al. (2012)^30^	Yes	The Jackson Laboratories	Yes	Sca-1, MHC-I, CD 29, CD 44, CD 90	Myeloid and hematopoietic markers	Not reported	Not reported	Not reported
Wei et al. (2015)^39^	Yes	No	Yes	CD 105, CD 73	CD 34, CD 45	Not reported	DMEM, 15% FBS	3–5
Xia et al. (2010)^25^	Yes	No	Yes	CD 29, CD 44, CD 105	CD 34, CD 45	Osteoblasts, chondrocytes, adipocytes	DMEM, 20% FBS, penicillin streptomycin, L-glutamine	10
Yang et al. (2020)^28^	No (only plastic adherence)	No	Yes	Not reported	Not reported	Not reported	Not reported	Not reported
Zhang et al. (2014)^26^	Yes	No	Yes	CD 73, CD 105, CD 90	CD 14, CD 34, CD 45, CD 79a	Not reported	StemPro MSCSserum-free medium, 10% FBS, penicillin, streptomycin	3
Zhou et. al. (2015)^27^	Yes	Chongqing stem cell bank	Yes	HLA-ABC, CD 29, CD 44, CD 90, CD 105	HLA-DR, CD 34, CD 45	Neural differentiation	DMEM/F12, 10% FBS, penicillin, streptomycin	5–10

### Risk of bias

The risk of bias was assessed using the SYRCLE Risk of Bias Tool for all 20 studies that met inclusion criteria (Table 3). Only three studies assessed and compared the relevant baseline characteristics including sex, age, weight, and lesion size.^35,37,41^ As the distribution was balanced for the intervention and the control group, these studies were rated with a “low” risk of bias for this domain. All the other studies were evaluated as “unclear” as it was not clearly stated if baseline characteristics were equally distributed between the groups. Despite 16 studies stating that the allocation of animals to experimental and control groups was random, only one^28^ of the studies explicitly described a method of random sequence generation. Therefore, all studies except one were judged as “unclear” in the domain of random sequence generation. Further none of the studies adequately described the method used to conceal allocation. None of the studies reported random housing nor sufficient information about the blinding of the investigators regarding the intervention. Ten studies^25,29–32,35–37,39,40^ mentioned blinding in terms of outcome assessments. Only six studies^29–32,36,37^ reported blinding of the investigators for all outcome assessments and were therefore judged with a “low” risk of bias. As none of the studies stated information about missing data and it was not obvious if all animals were included in the analysis, the domain of incomplete outcome data was rated as “unclear” for all studies. Similarly, in none of the studies, the study protocol was available, so it was unclear if the study was free of selective outcome reporting. In six studies^26,29,32,35,36,40^ no conflict of interest statement was reported despite funding. Therefore, these studies were rated with a “high” risk of bias in the domain of other sources of bias. As the possibility of bias could not be excluded in the other studies and therefore was rated as “unclear”. In one study^24^ ethical approval was not reported.

**Table 3:** Risk of bias assessment.

	1. Selection bias–Sequence generation	2. Selection bias–Baseline characteristics	3. Selection bias– Allocation concealment	4. Performance bias–Random housing	5. Performance bias–Blinding	6. Detection bias–Random outcome	7. Detection bias–Blinding	8. Attrition bias–Incomplete outcome data	9. Reporting bias–Selective outcome reporting	10. Other–Other sources of bias	Blinding mentioned	Randomization mentioned	Sample size calculation	Ethical approval mentioned	Conflict of interest statement mentioned
Cho et al. (2016)^34^	?	?	?	?	?	?	?	?	?	?	No	Yes	No	Yes	Yes
Ding et al. (2015)^22^	?	?	?	?	?	?	?	?	?	?	No	Yes	No	Yes	Yes
Ding et al. (2017)^23^	?	?	?	?	?	?	?	?	?	?	No	Yes	No	Yes	Yes
Larpthaveesarp et al. (2021)^37^	?	Low	?	?	?	?	Low	?	?	?	Yes	Yes	Yes	Yes	Yes
Kim et al. (2012)^35^	?	Low	?	?	?	?	?	?	?	High	Yes	Yes	No	Yes	No
Lee et al. (2010)^36^	?	?	?	?	?	?	Low	?	?	High	Yes	Yes	No	Yes	No
Li et al. (2014)^24^	?	?	?	?	?	?	?	?	?	?	No	Yes	No	No	Yes
Ohshima et al. (2015)^40^	?	?	?	?	?	?	?	?	?	High	Yes	Yes	No	Yes	No
Sakai et al. (2018)^41^	?	Low	?	?	?	?	?	?	?	?	No	Yes	No	Yes	Yes
van Velthoven et al. (2017)^38^	?	?	?	?	?	?	?	?	?	?	No	Yes	No	Yes	Yes
van Velthoven et al. (2013)^33^	?	?	?	?	?	?	?	?	?	?	No	No	No	Yes	Yes
van Velthoven et al. (2010)^29^	?	?	?	?	?	?	Low	?	?	?	Yes	Yes	No	Yes	No
van Velthoven et al. (2010)^31^	?	?	?	?	?	?	Low	?	?	High	Yes	No	No	Yes	No
van Velthoven et al. (2010)^32^	?	?	?	?	?	?	Low	?	?	High	Yes	No	No	Yes	No
van Velthoven et al. (2012)^30^	?	?	?	?	?	?	Low	?	?	?	Yes	Yes	No	Yes	Yes
Wei et al. (2015)^39^	?	?	?	?	?	?	?	?	?	?	Yes	Yes	Yes	Yes	Yes
Xia et al. (2010)^25^	?	?	?	?	?	?	?	?	?	?	Yes	Yes	No	Yes	Yes
Yang et al. (2020)^28^	Low	?	?	?	?	?	?	?	?	?	No	Yes	No	Yes	Yes
Zhang et al. (2014)^26^	?	?	?	?	?	?	?	?	?	High	No	Yes	No	Yes	No
Zhou et al. (2015)^27^	?	?	?	?	?	?	?	?	?	?	No	No	No	Yes	Yes

? = unclear risk of bias; Low = low risk of bias; High = high risk of bias.

### Effects of the interventions

#### Primary outcomes

All studies but one^40^ assessed a functional outcome. The sensorimotor outcome was measured most frequently (*n* = 15), followed by the cognitive outcome (*n* = 6),^22,24,26–28,37^ and only one study^39^ assessed participation in form of social interaction. Table 4 shows the list of the primary outcomes reported by each study. Meta-analysis of the animal studies was deemed feasible for the cylinder rearing test and the water maze test.

**Table 4:** Primary outcomes of the included studies.

Reference	Functional outcome						Participation		Survival of the animals
	Motor performance		Cognitive performance		Sensory function		Social interaction		
	Test	↑↓↔	Test	↑↓↔	Test	↑↓↔	Test	↑↓↔	
Cho et al. (2016);^34^ Korea	Rotarod test	2 weeks post MSC: ↔; 8 weeks post MSC: ↑ only MSC-EE group	NR		NR		NR		NR
	Grip strength test	2 weeks post MSC: ↔; 8 weeks post: ↑ only MSC-EE group							
Ding et al. (2015)^22^	NR		Morris water maze	21 days post injury: **↑**	NR		NR		NR
Ding et al. (2017)^23^	Hanging wire	16, 22, and 28 days post injury: ↑	NR		NR		NR		NR
	Vertical pole test	16, 22, and 28 days post injury: ↑							
Kim et al. (2012);^35^ Korea	Rotarod test	26 and 27 days post injury: ↔; 28 days post injury: ↑	NR		NR		NR		Survival at 28 days post injury; Sham control group =100%; MCAO-MC (vehicle) group = 40%; MCAO-MM (MSCs) group = 83%
	Cylinder test	28 days post injury: ↔							
Larpthaveesarp et al. (2021)^37^	Cylinder test	P61, P62 postnatal:; **↑** MACO+7 d MSC; ↔ MACO+3 d MSC	Novel object recognition	P54: **↔ ↑**only for MSC/EPO and EPO					MCAO+Vehicle group = 89%; 3 d MCAO+MSCs = 100%; 7 d MCAO+MSCs = 80%
			Open field test	P59: **↔**					
Lee et al. (2010);^36^ Korea	Rotarod test	14, 20, 30, and 40 days post MSCs: ↔	NR		NR		NR		NR
	Cylinder test	Overall: ↑; 14 days post MSCs: ↔; 20 days post MSCs: ↑							
Li et al. (2014);^24^ China			Shuttle box test	1–5 days post MSCs: **↔**	NR		NR		NR
Ohshima et al. (2015);^40^ Japan	NR		NR		NR		NR		NR
Sakai et al. (2018);^41^ Japan	Beam walk test	25 days post MSCs: ↑	NR		NR		NR		NR
van Velthoven et al. (2017);^38^ USA	Cylinder rearing test	28 days post injury: ↑	NR		NR		NR		The survival rate at the end of the study is unclear; before the randomization of the treatment groups and 3 days after tMCAO the survival rate is reported to be 87%
van Velthoven et al. (2013);^33^ Netherlands	Cylinder rearing test	14, 21, and 28 days post injury: ↑ at all timepoints	NR		NR		NR		Unclear by the end of the study; 87% post-MCAO and prior to randomization to the treatment groups
	Adhesion removal test	28 days post injury: ↑							
van Velthoven et al. (2010);^29^ Netherlands	Cylinder rearing test	10, 21, and 18 days post injury: ↑ MSC-3: versus VEH; 21 and 28 days post injury: ↑ MSCS-3+10: versus VEH, versus MSC-3	NR		NR		NR		Approx. 90% post HI insult
	Rotarod treadmill	21 days post injury: ↑MSC-3, MSC-3+10 versus VEH 28 days: ↑MSC-3, MSC-3+10 versus VEH↑ MSC-3+10 versus MSC-3							
van Velthoven et al. (2010);^31^ Netherlands	Cylinder rearing test	21 and 28 days post injury: ↑ versus VEH	NR		NR		NR		Approx. 90% post HI insult
van Velthoven et al. (2010);^32^ Netherlands	Cylinder rearing test	10 and 21 days post injury: ↑ versus VEH	NR		NR		NR		Approx. 90% post HI insult
van Velthoven et al. (2012);^30^ Netherlands	Cylinder rearing test	21 and 28 days post injury: ↑ versus VEH	NR		NR		NR		NR
Wei et al. (2015);^39^ USA	Adhesion removal test	10, 17, and 24 days post injury: ↑ versus saline	NR		Modified buried food-finding test	17 and 24 days after injury: ↑	Social interaction test	17 and 24 days after injury: ↑ versus Saline	NR
							Home-cage activity	17 and 24 days after injury: ↑ versus saline	
Xia et al. (2010);^25^ China	Modified neurological severity score: muscle status, abnormal movement Beam balance test reflex absence	14, 21, and 20 days post MSCs: ↑ overall score	NR		Modified neurological severity score: sensory tests	14, 21, and 20 days after MSCs: ↑overall score	NR		NR
Yang et al. (2020)^28^	NR		Spatial version of the Morris water maze task	4 weeks post injury: ↑	NR		NR		NR
Zhang et al. (2014);^26^ China	Longa scoring	6 h and 7 and 20 days post injury: ↑	Morris water maze task	4 weeks post injury: ↑ MSC-24 h versus MSC-72 h and VEH-24 h	NR		NR		NR
	Rotarod testing	20 days post injury: ↑							
Zhou et al. (2015)^27^	NR		Morris water maze test; object-in-place task	4 weeks post MSC:↑; 6 weeks old animals: ↑	NR		NR		NR

*NR* not reported, ↑ significant increase in performance, ↓ significant decrease in performance, ↔ no significant change in performance compared to control group, *MSC* mesenchymal stem cell administration, *MCAO* middle cerebral artery occlusion.

#### Sensorimotor outcome

**Fig. 2:**
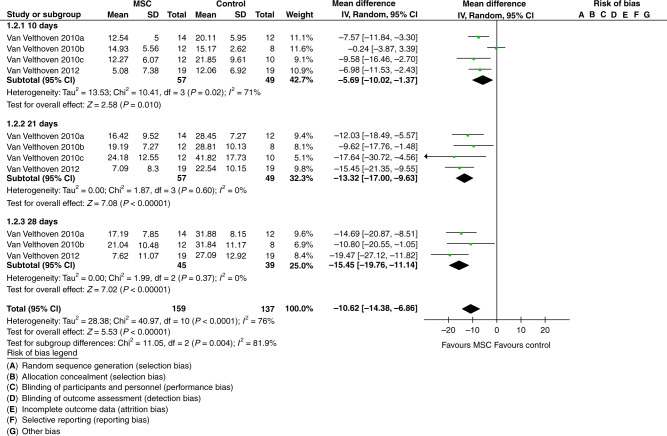
Effect of treatment with MSC compared to control (PAIS with no MSC) for cylinder rearing test at 10, 21, and 28 days after PAIS. Difference in route of application and dose.

The cylinder rearing test was most often used to assess the motor outcome (*n* = 9).^29–33,35–38^ For four of these studies,^29–32^ we were able to conduct a meta-analysis based on their comparability including PAIS mode, species, and type of MSCs. The meta-analysis shows a significant improvement in favor of the MSCs group (MD: −10.62; 95% CI: −14.38 to −6.86) for all test days (10, 21, and 28 days) compared to the control group (Fig. 2). However, the heterogeneity at 10 days is high (*I*^2^ = 71%) but not for days 21 and 28 (*I*^2^ = 0%). The biggest difference between MSCs and control group is observable on day 28 (MD:−15.45; 95% CI: −19.76 to −11.14).

The rotarod test was the second most reported motor outcome (*n* = 5).^26,29,34–36^ Due to the heterogeneity among these studies, no meta-analysis was possible. Three studies^26,29,35^ reported a significant improvement in performance on rotarod in the MSCs group compared to the control group. Furthermore, a second dose of MSCs on day 10 increased the motor performance compared to a single application of MSCs.^29^

Further tests that were used to test the motor performance were the beam walk test (*n* = 2),^25,41^ the adhesion removal test (*n* = 2),^33,39^ the grip strength test (*n* = 1),^34^ the hanging wire test (*n* = 1),^23^ the vertical pole test (*n* = 1),^23^ and longa scorning (*n* = 1).^26^ Only one study using the grip strength test could detect no improved motor performance for the MSCs group.

#### Cognitive outcome

**Fig. 3:**
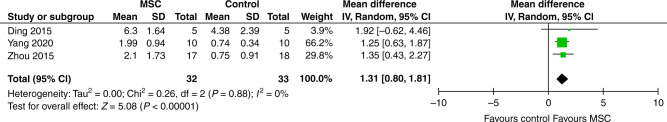
Effect of treatment with MSC compared to control (PAIS with no MSC) for water maze test. Difference in route of application and dose.

Four studies^22,26–28^ investigated cognitive performance with the water maze test being the most commonly applied. The novel object recognition test was used in one study,^37^ as well as the shuttle box test,^24^ open field test,^37^ and object-in-place test.^27^ For three studies,^22,27,28^ it was possible to pool the results of the water maze test. The water maze performance was improved by 1.31 MD (95% CI: 0.80 to 1.81) with no heterogeneity (*I*^2^ = 0%) in favor of the MSCs group (Fig. 3).

#### Sensory function

Two studies^25,39^ reported measuring sensory function. One study^39^ assessed the olfactory function with the modified buried food-finding test at 17 days. They could detect a significant improvement measured by less time to find food for the stroke animals that received MSCs. The other study reported several sensory functions within a modified neurological severity score including as well motor tests. The overall test battery showed significantly better results for the MSCs group compared to the control group.

#### Participation

Only one study^39^ tested social interaction using social interaction tests and home-cage activity. The results of this study demonstrated better social behavior in PAIS animals treated with MSCs compared to the control group animals.

#### Survival/mortality

Seven studies^29,31–33,35,37,38^ reported on the survival of the animals. Two studies^37,38^ reported the survival rate for the different groups of animals. Both studies reported a survival rate of 87% only post injury, not at the end of the study.

### Secondary outcomes

Secondary outcomes are listed in the Supplementary material and include lesion size; markers for neurogenesis, apoptosis, neuronal development; markers for inflammation; and distribution of MSCs.

## Discussion

This is the first systematic review and meta-analysis of preclinical studies investigating the effects of MSCs in an experimental model of PAIS. The main finding is that MSC treatment favors sensorimotor and cognitive performance in PAIS-injured animals compared to vehicle-treated animals.

### Primary outcomes

The primary outcomes assessed in this review included the functional outcome, as PAIS often leads to functional deficits such as cerebral palsy, cognitive deficits, and neurodevelopmental delay that may result in reduced physical activity and participation in later life.^42^ There was a large array of measurements being used to assess functional outcomes including 16 different tests in included studies. The lack of standardization in outcome measurement has been addressed in a recent review suggesting greater consistency in choice, application, and reporting of outcomes.^19^ We found that sensorimotor outcome was measured most frequently (*n* = 15), followed by the cognitive outcome (*n* = 6) and only one study assessed participation in form of social interaction.

We considered a meta-analysis for the cylinder rearing test, a test for the sensorimotor outcome, and the water maze Morris test, a test for the cognitive outcome, to be feasible because these studies were comparable, including PAIS mode, species, and type of MSCs. We found that MSCs significantly improved sensorimotor and cognitive performances are consistent with other reviews that analyzed the effect of stem cell therapy in neonatal animal models of HIE, including the same injury models as for PAIS.^11,12^ Despite high heterogeneity for functional outcomes (14 studies were analyzed for sensorimotor and 5 for cognitive outcomes) in the review of Archambault et al. the data of our meta-analysis are in agreement with their data, showing the benefit of MSCs treatment following PAIS.^11^ As well, the recently published comprehensive review of Serrenho et al. on stem cell therapy for HIE found that 80% of all studies (*n* = 58) improved either cognitive or motor outcome or both.^12^ Overall, this was also evident in most of the studies included in this review showing the beneficial effects of MSCs.

While the most common approach for modeling PAIS reported by Faustino-Medes et al. is the transient unilateral ligation of the common carotid artery followed by hypoxia,^43^ other studies stated that the lesion created by a single permanent artery occlusion is more similar to the lesion that we can have in PAIS.^44,45^ Overall, we could identify four different types of injury: (1) transient ligation + hypoxia (8% O_2_) for 30 min, 90 min, 2 h, 2.5 h, 3,5 h; (2) transient ligation + hypoxia (10% O_2_) for 45 min; (3) permanent ligation without hypoxia; and (4) ligation with reperfusion. In addition, distinct variations of these four types have been described and studied in the literature, each owing to differences in lesion size, clinical features, and underlying processes.^43–47^ As animal models are considered of crucial importance to explore mechanisms underlying the disease and are supposed to replicate and assess the safety and efficacy of treatments, the choice of animal models may be of great importance when studying the effect of MSCs. Since we were only able to conduct a meta-analysis on one injury group (ligation + hypoxia), it is not clear to what extent different models influence the outcome.

Mortality and adverse outcomes are important endpoints; however, in MSCs-based preclinical studies, they are reported barely. Only seven studies included in this review stated the data on the survival of animals, and just two of these studies described the survival data based on groups. The other studies reported the survival rate following the induction of PAIS but before the initiation of treatment. The finding highlights the importance to report the survival rate in animal studies, as well as the initial and final number of animals included in the studies. None of the included studies stated if adverse outcomes occurred. Notably, all clinical phase I trials reported yet on MSCs-based therapy did not result in adverse events in severe IVH,^48^ HIE,^49–51^ and preterm infants with risk for BPD.^52^ Specifically for the condition of PAIS, a phase I trial has been completed very recently in the Netherlands and no adverse events were described.^53^ However, no studies have been completed to evaluate the efficacy of stem cell therapy in neonates.^54,55^ Furthermore, all the studies are rather short term, and long-term follow-up is needed to reassure the safety of MSCs in the long term.

### Secondary outcomes

Our secondary outcomes included inflammation markers for the brain, lesion size and outcomes, and/or markers for neurogenesis, apoptosis, and neuronal development. Neuroimaging studies found beneficial results regarding lesion size in the MSCs group. Most IHC studies reported improvement in pathological changes in animals receiving MSCs treatment. Several studies reported increased neurogenesis after the application of MSCs, for different timepoints and brain regions, and enhanced synaptic plasticity. Studies on white matter injury showed an increase in BrdU/Olig2 cells, a decrease in MAP2 and MBP loss, increased MPB optical density, and lateral arborization in animals with PAIS treated with MSCs. The effects on angiogenesis, astrogliosis, and pro-inflammatory cytokines were unclear.

### Heterogeneity among included studies

Overall, we observed a high heterogeneity among studies on cell source, cell administration, the timing of administration after injury, cell number administered, and sex of the animals, when reported.

Bone marrow was the most common source of MSCs (*n* = 11). Further MSCs were derived from human umbilical cord blood (*n* = 3), placenta (*n* = 2), Wharton’s jelly (*n* = 1), and adipose tissue (*n* = 1). Two studies did not specify or report the source of MSCs. Although MSCs from different tissues display similar immunophenotypic patterns, many studies demonstrated differences in marker expression.^56^ Liau et al. recently highlighted therapeutic benefits for MSCs obtained from umbilical cord tissue due to their availability and immune evasive nature.^57^

We found five different forms of application with an intracerebral injection of MSCs as the most common route of delivery (*n* = 10). Two studies compared two routes of application, intravenous versus intraperitoneal, and reported a slight benefit for intravenous application.^26,40^ Overall, it is commonly believed that local (e.g., intraventricular) rather than systemic (e.g., intravenous or intraperitoneal) stem cell delivery is therapeutically more effective.^58^ The intranasal delivery of MSCs seems an optimal route of administration in terms of non-invasiveness and practicability and is considered an effective path for cell-based therapies.^59^

Another essential issue in clinical translation is the optimal timing for MSCs application. We found that most of the studies administered MSCs three days after PAIS (*n* = 8). Two studies compared an earlier versus later application of MSCs with more beneficial effects for the earlier application.^26,37^ Although the question has not yet been fully clarified, some studies also suggest that a later application of stem cells weakens the effect.^8,58^

In terms of doses, we found that most often 1 × 10^6^ cells were used (*n* = 7). A cell dose of 5 × 10^6^ and 10^7^ cells/kg has been described as safe in the short term in the first clinical studies using MSCs in neonatal diseases.^48,51,52,60,61^ In general, higher doses of MSCs are considered more effective but the upper limit of MSCs has not been defined so far.^8^ Furthermore, Ahn et al. concluded that the optimal doses of MSCs for the best therapeutic effects should be determined based on the timing and route of MSCs transplantation.^58^

Only half of our studies reported the sex of the animals, three studies used male pups, and seven studies pups of both sexes. None of the included studies investigated the effect of MSCs in females alone, nor did comparative analysis. To date, several studies have shown that perinatal stroke appears to be sex-dependent and may also influence the effect of stem cell treatment.^62–64^ The male sex has a higher vulnerability, possibly due to neuroinflammation, oxidative stress, and cell death pathways.^63^ Due to these existing differences, studies must take sex into account in the study design and, above all, report on it.

### Quality of the studies

Overall, the included trials were characterized by high or unclear risk of bias in most domains of the SYRCLE risk of bias tool, and imprecision of the estimates. None of the studies reported on allocation concealment, random housing, blinding of the caregivers, or random outcome assessment. Only one study reported on sequence generation,^28^ three studies balanced relevant baseline characteristics adequately, and five studies reported on blinding of the outcome assessors. A study protocol was not available for any of the animal studies. This leads to an unclear risk of reporting bias and poor transparency in general. The problem of unclear risk of bias has already been highlighted in other systematic reviews of preclinical studies on neonatal pathological conditions.^10,11,65^ Estimates of the effect size were imprecise for most outcomes, due to few and small studies reporting the same outcome measures, and wide confidence of intervals.

### Strengths and limitations

The strengths of our systematic review include a rigorous peer-reviewed search strategy, the registration of a protocol before screening and analyzing the studies, and the use of international guidelines and standards to conduct our systematic review.

However, our review was limited by the fact that a large number of published data were available only in the form of figures and not in an easily extractable numerical form. Thus, most of all the data we used were extracted from figures; minor distortion of data is possible, but all groups would be equally affected. An additional limitation in this review is the choice of the primary outcomes, limited to functional outcome parameters. To include histological benefits of MSC treatment, such as the size or volume of the brain lesion, would have increased the translational value of the findings, also considering that the behavioral test was conducted in the first weeks of life. Finally, the study design of the included studies presented relevant differences in the model of inducing PAIS and MSCs, thus causing heterogeneity and inconsistency, which affect the overall certainty of evidence.

## Conclusion

Preclinical studies suggest that MSCs treatment might improve sensorimotor and cognitive performance in PAIS-injured neonatal animals. However, the quality of the evidence is low because of study limitations and imprecision of the estimates. Confirmatory studies on MSCs for PAIS should pre-register the study protocol, use an appropriate sample size based on a relevant outcome, and measures to minimize bias should be considered.

### Supplementary information

PRISMA_2020_abstract_checklist

PRISMA_2020_checklist_(1)

Supplementary material – Table

Supplementary material – Search Strategies

Supplementary material – secondary outcomes

## Data Availability

The datasets generated during and/or analyzed during the current study are available from the corresponding author on reasonable request.
