# Supplementary material for: Mesenchymal stem cell therapy in perinatal arterial ischemic stroke: systematic review of preclinical studies
Source: Pediatr Res. 2022 Jul 29;95(1):18–33. doi: 10.1038/s41390-022-02208-3 (PMC10798891; doi:10.1038/s41390-022-02208-3)
Supplement: Supplementary file 4 — Supplementary material – Search Strategies [file 41390_2022_2208_MOESM4_ESM.pdf]

Supplementary material - Search Strategies

1.1 PubMed

Date of search 20210219

No language restriction or publication date restriction was used

#1 ("animal experimentation"[MeSH Terms] OR "models, animal"[MeSH Terms] OR"Animals"[Mesh:noexp] OR "animal population groups"[MeSH Terms] OR "vertebrates"[MeSH Terms:noexp] OR "mammals"[MeSH Terms:noexp] OR "primates"[MeSH Terms:noexp] OR "artiodactyla"[MeSH Terms] OR "carnivora"[MeSH Terms] OR "cetacea"[MeSH Terms] OR "chiroptera"[MeSH Terms] OR "elephants"[MeSH Terms] OR "hyraxes"[MeSH Terms] OR "insectivora"[MeSH Terms] OR "lagomorpha"[MeSH Terms] OR "marsupialia"[MeSH Terms] OR "monotremata"[MeSH Terms] OR "perissodactyla"[MeSH Terms] OR "rodentia"[MeSH Terms] OR "scandentia"[MeSH Terms] OR "sirenia"[MeSH Terms] OR "xenarthra"[MeSH Terms] OR "haplorhini"[MeSH Terms:noexp] OR "strepsirhini"[MeSH Terms] OR "platyrrhini"[MeSH Terms] OR "tarsii"[MeSH Terms] OR "catarrhini"[MeSH Terms:noexp] OR "cercopithecidae"[MeSH Terms] OR "hylobatidae"[MeSH Terms] OR "hominidae"[MeSH Terms:noexp] OR "gorilla gorilla"[MeSH Terms] OR "pan paniscus"[MeSH Terms] OR "pan troglodytes"[MeSH Terms] OR "pongo pygmaeus"[MeSH Terms]) OR ((animals[tiab] OR animal[tiab] OR mice[Tiab] OR mus[Tiab] OR mouse[Tiab] OR murine[Tiab] OR woodmouse[tiab] OR rats[Tiab] OR rat[Tiab] OR murinae[Tiab] OR muridae[Tiab] OR cottonrat[tiab] OR cottonrats[tiab] OR hamster[tiab] OR hamsters[tiab] OR cricetinae[tiab] OR rodentia[Tiab] OR rodent[Tiab] OR rodents[Tiab] OR pigs[Tiab] OR pig[Tiab] OR swine[tiab] OR swines[tiab] OR piglets[tiab] OR piglet[tiab] OR boar[tiab] OR boars[tiab] OR "sus scrofa"[tiab] OR ferrets[tiab] OR ferret[tiab] OR polecat[tiab] OR polecats[tiab] OR "mustela putorius"[tiab] OR "guinea pigs"[Tiab] OR "guinea pig"[Tiab] OR cavia[Tiab] OR callithrix[Tiab] OR marmoset[Tiab] OR marmosets[Tiab] OR cebuella[Tiab] OR hapale[Tiab] OR octodon[Tiab] OR chinchilla[Tiab] OR chinchillas[Tiab] OR gerbillinae[Tiab] OR gerbil[Tiab] OR gerbils[Tiab] OR jird[Tiab] OR jirds[Tiab] OR merione[Tiab] OR meriones[Tiab] OR rabbits[Tiab] OR rabbit[Tiab] OR hares[Tiab] OR hare[Tiab] OR cats[Tiab] OR cat[Tiab] OR carus[Tiab] OR felis[Tiab] OR dogs[Tiab] OR dog[Tiab] OR canine[Tiab] OR canines[Tiab] OR canis[Tiab] OR sheep[Tiab] OR sheeps[Tiab] OR mouflon[Tiab] OR mouflons[Tiab] OR ovis[Tiab] OR goats[Tiab] OR goat[Tiab] OR capra[Tiab] OR capras[Tiab] OR rupicapra[Tiab] OR rupicapras[Tiab] OR chamois[Tiab] OR haplorhini[Tiab] OR monkey[Tiab] OR monkeys[Tiab] OR anthropoidea[Tiab] OR anthropoids[Tiab] OR saguinus[Tiab] OR tamarin[Tiab] OR tamarins[Tiab] OR leontopithecus[Tiab] OR hominidae[Tiab] OR ape[Tiab] OR apes[Tiab] OR "pan paniscus"[Tiab] OR bonobo[Tiab] OR bonobos[Tiab] OR gibbon[Tiab] OR gibbons[Tiab] OR siamang[Tiab] OR siamangs[Tiab] OR nomascus[Tiab] OR symphalangus[Tiab] OR chimpanzee[Tiab] OR chimpanzees[Tiab] OR prosimian[Tiab] OR prosimians[Tiab] OR "bush baby"[Tiab] OR bush babies[Tiab] OR galagos[Tiab] OR galago[Tiab] OR pongidae[Tiab] OR gorilla[Tiab] OR gorillas[Tiab] OR "pongo pygmaeus"[Tiab] OR orangutan[Tiab] OR orangutans[Tiab] OR lemur[Tiab] OR lemurs[Tiab] OR lemuridae[Tiab] OR horse[Tiab] OR horses[Tiab] OR equus[Tiab] OR cow[Tiab] OR calf[Tiab] OR bull[Tiab] OR sciuridae[Tiab] OR squirrel[Tiab] OR

squirrels[Tiab] OR chipmunk[Tiab] OR chipmunks[Tiab] OR suslik[Tiab] OR susliks[Tiab] OR vole[Tiab] OR voles[Tiab] OR lemming[Tiab] OR lemmings[Tiab] OR muskrat[Tiab] OR muskrats[Tiab] OR lemmus[Tiab] OR otter[Tiab] OR otters[Tiab] OR marten[Tiab] OR martens[Tiab] OR martes[Tiab] OR weasel[Tiab] OR badger[Tiab] OR badgers[Tiab] OR ermine[Tiab] OR mink[Tiab] OR minks[Tiab] OR sable[Tiab] OR sables[Tiab] OR gulo[Tiab] OR gulos[Tiab] OR wolverine[Tiab] OR wolverines[Tiab] OR mustela[Tiab] OR llama[Tiab] OR llamas[Tiab] OR alpaca[Tiab] OR alpacas[Tiab] OR camelid[Tiab] OR camelids[Tiab] OR guanaco[Tiab] OR guanacos[Tiab] OR chiroptera[Tiab] OR chiropteras[Tiab] OR bat[Tiab] OR bats[Tiab] OR fox[Tiab] OR foxes[Tiab] OR donkey[Tiab] OR donkeys[Tiab] OR mule[Tiab] OR mules[Tiab] OR zebra[Tiab] OR zebras[Tiab] OR shrew[Tiab] OR shrews[Tiab] OR bison[Tiab] OR bisons[Tiab] OR buffalo[Tiab] OR buffaloes[Tiab] OR deer[Tiab] OR deers[Tiab] OR bear[Tiab] OR bears[Tiab] OR panda[Tiab] OR pandas[Tiab] OR "wild hog"[Tiab] OR "wild boar"[Tiab] OR fitchew[Tiab] OR fitch[Tiab] OR beaver[Tiab] OR beavers[Tiab] OR jerboa[Tiab] OR jerboas[Tiab] OR capybara[Tiab] OR capybaras[Tiab] OR canine [tiab] OR bovine [tiab] OR porcine [tiab] OR hog [tiab] OR hogs [tiab]) NOT medline[sb])

7151599 records

#2 ((("stroke"[MeSH Terms]) OR (stroke[Title/Abstract])) OR (perinatal stroke[Title/Abstract] OR arterial ischemic stroke[Title/Abstract] OR AIS[Title/Abstract] OR PAIS[Title/Abstract] OR neonatal stroke[Title/Abstract])) OR (perinatal arterial ischemic stroke[Title/Abstract] OR neonatal arterial ischemic stroke[Title/Abstract] OR neonatal ischemic stroke[Title/Abstract] OR perinatal ischemic stroke[Title/Abstract])

304530 records

#3 (("mesenchymal stem cells"[MeSH Terms]) OR ("mesenchymal stem cell transplantation"[MeSH Terms])) OR (Mesenchymal stem cells OR mesenchymal stromal cells OR MSC[Title/Abstract] OR MSCs OR BMSC*[Title/Abstract] OR ADSC*[Title/Abstract] OR UC-MSC*[Title/Abstract] OR UCB-MSC*[Title/Abstract])

81368 records

#4 #1 AND #2 AND #3

818 records

1.2 Embase (Elsevier)

Date of search 20210219

No language restriction or publication date restriction was used

#1 'animal experiment'/exp OR 'animal experiment' OR 'animal model'/exp OR 'animal model' OR 'experimental animal'/exp OR 'experimental animal' OR 'transgenic animal'/exp OR 'transgenic animal' OR 'male animal'/exp OR 'male animal' OR 'female animal'/exp OR 'female animal' OR 'juvenile animal'/exp OR 'juvenile animal' OR 'animal'/exp OR 'animal'

OR 'vertebrate'/exp OR 'vertebrate' OR 'mammal'/exp OR 'mammal' OR 'therian'/exp OR 'therian' OR 'monotreme'/exp OR 'monotreme' OR 'placental mammal'/exp OR 'placental mammal' OR 'marsupial'/exp OR 'marsupial' OR 'euarchontoglires'/exp OR 'euarchontoglires' OR 'afrotheria'/exp OR 'afrotheria' OR 'boreoeutheria'/exp OR 'boreoeutheria' OR 'laurasiatheria'/exp OR 'laurasiatheria' OR 'xenarthra'/exp OR 'xenarthra' OR 'primate'/exp OR 'primate' OR 'dermoptera'/exp OR 'dermoptera' OR 'glires'/exp OR 'glires' OR 'scandentia'/exp OR 'scandentia' OR 'haplorhini'/exp OR 'haplorhini' OR 'prosimian'/exp OR 'prosimian' OR 'simian'/exp OR 'simian' OR 'tarsiiform'/exp OR 'tarsiiform' OR 'catarrhini'/exp OR 'catarrhini' OR 'platyrrhini'/exp OR 'platyrrhini' OR 'ape'/exp OR 'ape' OR 'cercopithecidae'/exp OR 'cercopithecidae' OR 'hominid'/exp OR 'hominid' OR 'hylobatidae'/exp OR 'hylobatidae' OR 'chimpanzee'/exp OR 'chimpanzee' OR 'gorilla'/exp OR 'gorilla' OR 'orangutan'/exp OR 'orangutan' OR animal:ti,ab OR animals:ti,ab OR shrews:ti,ab OR sorex:ti,ab OR araneus:ti,ab OR crocidura:ti,ab OR russula:ti,ab OR 'european mole':ti,ab OR talpa:ti,ab OR chiroptera:ti,ab OR bat:ti,ab OR bats:ti,ab OR eptesicus:ti,ab OR serotinus:ti,ab OR myotis:ti,ab OR dasycneme:ti,ab OR daubentonii:ti,ab OR pipistrelle:ti,ab OR pipistrellus:ti,ab OR cat:ti,ab OR cats:ti,ab OR felis:ti,ab OR catus:ti,ab OR feline:ti,ab OR dog:ti,ab OR dogs:ti,ab OR canis:ti,ab OR canine:ti,ab OR canines:ti,ab OR otter:ti,ab OR otters:ti,ab OR lutra:ti,ab OR badger:ti,ab OR badgers:ti,ab OR meles:ti,ab OR fitchew:ti,ab OR fitch:ti,ab OR foumart:ti,ab OR foulmart:ti,ab OR ferrets:ti,ab OR ferret:ti,ab OR polecat:ti,ab OR polecats:ti,ab OR mustela:ti,ab OR putorius:ti,ab OR weasel:ti,ab OR weasels:ti,ab OR fox:ti,ab OR foxes:ti,ab OR vulpes:ti,ab OR 'common seal':ti,ab OR phoca:ti,ab OR vitulina:ti,ab OR 'grey seal':ti,ab OR halichoerus:ti,ab OR horse:ti,ab OR horses:ti,ab OR equus:ti,ab OR equine:ti,ab OR equidae:ti,ab OR donkey:ti,ab OR donkeys:ti,ab OR mule:ti,ab OR mules:ti,ab OR pig:ti,ab OR pigs:ti,ab OR swine:ti,ab OR swines:ti,ab OR hog:ti,ab OR hogs:ti,ab OR boar:ti,ab OR boars:ti,ab OR porcine:ti,ab OR piglet:ti,ab OR piglets:ti,ab OR sus:ti,ab OR scrofa:ti,ab OR llama:ti,ab OR llamas:ti,ab OR lama:ti,ab OR glama:ti,ab OR deer:ti,ab OR deers:ti,ab OR cervus:ti,ab OR elaphus:ti,ab OR cow:ti,ab OR cows:ti,ab OR 'bos taurus':ti,ab OR 'bos indicus':ti,ab OR bovine:ti,ab OR bull:ti,ab OR bulls:ti,ab OR cattle:ti,ab OR bison:ti,ab OR bisons:ti,ab OR sheep:ti,ab OR sheeps:ti,ab OR 'ovis aries':ti,ab OR ovine:ti,ab OR lamb:ti,ab OR lambs:ti,ab OR mouflon:ti,ab OR mouflons:ti,ab OR goat:ti,ab OR goats:ti,ab OR capra:ti,ab OR caprine:ti,ab OR chamois:ti,ab OR rupicapra:ti,ab OR leporidae:ti,ab OR lagomorpha:ti,ab OR lagomorph:ti,ab OR rabbit:ti,ab OR rabbits:ti,ab OR oryctolagus:ti,ab OR cuniculus:ti,ab OR laprine:ti,ab OR hares:ti,ab OR lepus:ti,ab OR rodentia:ti,ab OR rodent:ti,ab OR rodents:ti,ab OR murinae:ti,ab OR mouse:ti,ab OR mice:ti,ab OR mus:ti,ab OR musculus:ti,ab OR murine:ti,ab OR woodmouse:ti,ab OR apodemus:ti,ab OR rat:ti,ab OR rats:ti,ab OR rattus:ti,ab OR norvegicus:ti,ab OR 'guinea pig':ti,ab OR 'guinea pigs':ti,ab OR cavia:ti,ab OR porcellus:ti,ab OR hamster:ti,ab OR hamsters:ti,ab OR mesocricetus:ti,ab OR cricetulus:ti,ab OR cricetus:ti,ab OR gerbil:ti,ab OR gerbils:ti,ab OR jird:ti,ab OR jirds:ti,ab OR meriones:ti,ab OR unguiculatus:ti,ab OR jerboa:ti,ab OR jerboas:ti,ab OR jaculus:ti,ab OR chinchilla:ti,ab OR chinchillas:ti,ab OR beaver:ti,ab OR beavers:ti,ab OR 'castor fiber':ti,ab OR 'castor canadensis':ti,ab OR sciuridae:ti,ab OR squirrel:ti,ab OR squirrels:ti,ab OR sciurus:ti,ab OR chipmunk:ti,ab OR chipmunks:ti,ab OR marmot:ti,ab OR marmots:ti,ab OR marmota:ti,ab OR suslik:ti,ab OR susliks:ti,ab OR spermophilus:ti,ab OR cynomys:ti,ab OR cottonrat:ti,ab OR cottonrats:ti,ab OR sigmodon:ti,ab OR vole:ti,ab OR voles:ti,ab OR microtus:ti,ab OR myodes:ti,ab OR glareolus:ti,ab OR primate:ti,ab OR primates:ti,ab OR prosimian:ti,ab OR prosimians:ti,ab OR lemur:ti,ab OR lemurs:ti,ab OR lemuridae:ti,ab OR loris:ti,ab OR 'bush baby':ti,ab OR 'bush babies':ti,ab OR bushbaby:ti,ab OR bushbabies:ti,ab OR galago:ti,ab OR galagos:ti,ab OR anthropoidea:ti,ab OR anthropoids:ti,ab OR simian:ti,ab OR simians:ti,ab OR monkey:ti,ab OR monkeys:ti,ab OR marmoset:ti,ab OR

marmosets:ti,ab OR callithrix:ti,ab OR cebuella:ti,ab OR tamarin:ti,ab OR tamarins:ti,ab OR saguinus:ti,ab OR leontopithecus:ti,ab OR 'squirrel monkey':ti,ab OR 'squirrel monkeys':ti,ab OR saimiri:ti,ab OR 'night monkey':ti,ab OR 'night monkeys':ti,ab OR 'owl monkey':ti,ab OR 'owl monkeys':ti,ab OR douroucoulis:ti,ab OR aotus:ti,ab OR 'spider monkey':ti,ab OR 'spider monkeys':ti,ab OR ateles:ti,ab OR baboon:ti,ab OR baboons:ti,ab OR papio:ti,ab OR 'rhesus monkey':ti,ab OR macaque:ti,ab OR macaca:ti,ab OR mulatta:ti,ab OR cynomolgus:ti,ab OR fascicularis:ti,ab OR 'green monkey':ti,ab OR 'green monkeys':ti,ab OR chlorocebus:ti,ab OR vervet:ti,ab OR vervets:ti,ab OR pygerythrus:ti,ab OR hominoidea:ti,ab OR ape:ti,ab OR apes:ti,ab OR hylobatidae:ti,ab OR gibbon:ti,ab OR gibbons:ti,ab OR siamang:ti,ab OR siamangs:ti,ab OR nomascus:ti,ab OR symphalangus:ti,ab OR hominidae:ti,ab OR orangutan:ti,ab OR orangutans:ti,ab OR pongo:ti,ab OR chimpanzee:ti,ab OR chimpanzees:ti,ab OR 'pan troglodytes':ti,ab OR bonobo:ti,ab OR bonobos:ti,ab OR 'pan paniscus':ti,ab OR gorilla:ti,ab OR gorillas:ti,ab

29362382 records

#2 'cerebrovascular accident'/exp OR 'perinatal stroke'/exp OR 'neonatal stroke'/exp OR stroke:ab,ti OR 'perinatal stroke':ab,ti OR 'neonatal stroke':ab,ti OR pais:ab,ti OR ais:ab,ti OR 'arterial ischemic stroke':ab,ti OR 'neonatal arterial ischemic stroke':ab,ti OR 'perinatal arterial ischemic stroke':ab,ti OR 'neonatal ischemic stroke':ab,ti OR 'perinatal ischemic stroke':ab,ti

518710 records

#3 'mesenchymal stem cell'/exp OR 'mesenchymal stem cell transplantation'/exp OR 'mesenchymal stem cells':jt OR 'mesenchymal stromal cells':jt OR msc:jt OR 'mcs* OR bmsc*':jt OR adsc*:jt OR 'uc msc*':jt OR 'ucb msc*':jt

71591 records

#4 #1 AND #2 AND #3

1318 records

#5 #4 AND [embase]/lim NOT ([embase]/lim AND [medline]/lim)

532 records

1.3 Web of Science (Clarivate Analytics)

Date of search 20210219

No language restriction or publication date restriction was used

Databases= WOS, BIOSIS, CABI, FSTA, KJD, MEDLINE, RSCI, SCIELO, ZOOREC Timespan=All years

Search language=Auto

#1 TOPIC: ("animal experimentation" OR "models, animal" OR "Animals" OR "animal population groups" OR "vertebrates" OR "mammals" OR "primates" OR "artiodactyla"OR "carnivora"OR "cetacea"OR "chiroptera"OR "elephants"OR "hyraxes"OR "insectivora"OR "lagomorpha"OR "marsupialia"OR "monotremata"OR "perissodactyla"OR "rodentia"OR "scandentia"OR "sirenia"OR "xenarthra"OR "haplorhini"OR "strepsirhini"OR "platyrrhini"OR "tarsii"OR "catarrhini"OR "cercopithecidae"OR "hylobatidae"OR "hominidae"OR "gorilla gorilla"OR "pan paniscus"OR "pan troglodytes"OR "pongo pygmaeus" OR animals OR animal OR mice OR mus OR mouse OR murine OR woodmouse OR rats OR rat OR murinae OR Muridae OR cottonrat OR cottonrats OR hamster OR hamsters OR cricetinae OR rodentia OR rodent OR rodents OR pigs OR pig OR swine OR swines OR piglets OR piglet OR boar OR boars OR "sus scrofa"OR ferrets OR ferret OR polecat OR polecats OR "mustela putorius"OR "guinea pigs" OR "guinea pig" OR cavia OR Callithrix OR marmoset OR marmosets OR cebuella OR hapale OR octodon OR chinchilla OR chinchillas OR gerbillinae OR gerbil OR gerbils OR jird OR jirds OR merione OR meriones OR rabbits OR rabbit OR hares OR hare OR cats OR cat OR carus OR felis OR dogs OR dog OR canine OR canines OR canis OR sheep OR sheeps OR mouflon OR mouflons OR ovis OR goats OR goat OR capra OR capras OR rupicapra OR rupicapras OR chamois OR haplorhini OR monkey OR monkeys OR anthropoidea OR anthropoids OR saguinus OR tamarin OR tamarins OR leontopithecus OR hominidae OR ape OR apes OR "pan paniscus" OR bonobo OR bonobos OR gibbon OR gibbons OR siamang OR siamangs OR nomascus OR symphalangus OR chimpanzee OR chimpanzees OR prosimian OR prosimians OR "bush baby" OR bush babies OR galagos OR galago OR pongidae OR g ORilla OR g ORillas OR "pongo pygmaeus" OR orangutan OR orangutans OR lemur OR lemurs OR lemuridae OR horse OR horses OR equus OR cow OR calf OR bull OR sciuridae OR squirrel OR squirrels OR chipmunk OR chipmunks OR suslik OR susliks OR vole OR voles OR lemming OR lemmings OR muskrat OR muskrats OR lemmus OR otter OR otters OR marten OR martens OR martes OR weasel OR badger OR badgers OR ermine OR mink OR minks OR sable OR sables OR gulo OR gulos OR wolverine OR wolverines OR mustela OR llama OR llamas OR alpaca OR alpacas OR camelid OR camelids OR guanaco OR guanacos OR chiroptera OR chiropteras OR bat OR bats OR fox OR foxes OR donkey OR donkeys OR mule OR mules OR zebra OR zebras OR shrew OR shrews OR bison OR bisons OR buffalo OR buffaloes OR deer OR deers OR bear OR bears OR panda OR pandas OR "wild hog" OR "wild boar" OR fitchew OR fitch OR beaver OR beavers OR jerboa OR jerboas OR capybara OR capybaras OR canine OR bovine OR porcine OR hog OR hogs)

29,620,129 records

#2 TOPIC: (Stroke OR perinatal stroke OR arterial ischemic stroke OR AIS OR PAIS OR neonatal stroke OR perinatal arterial ischemic stroke OR neonatal arterial ischemic stroke OR neonatal ischemic stroke OR perinatal ischemic stroke)

596111 records

#3

TOPIC: (((Mesenchymal stem cells OR mesenchymal stromal cells OR mesenchymal stem cell therapy OR mesenchymal stromal cell therapy OR MSC*OR BMSC* OR ADSC* OR UC-MSC* OR UCB-MSC*))

138166 records

#4 #1 AND #2 AND #3

2028 records

3378 records in EndNote in total

EndNote indicates 589 of these as duplicates, = 2789 unique records.
