# Supplementary material for: Mesenchymal stem cell therapy in perinatal arterial ischemic stroke: systematic review of preclinical studies
Source: Pediatr Res. 2022 Jul 29;95(1):18–33. doi: 10.1038/s41390-022-02208-3 (PMC10798891; doi:10.1038/s41390-022-02208-3)
Supplement: Supplementary file 5 — Supplementary material – secondary outcomes [file 41390_2022_2208_MOESM5_ESM.pdf]

Supplementary material - Secondary Outcomes

Lesion size

Lesion size was analysed by neuroimaging and immunohistochemistry in four [30,35,38,41] and ten [22,23,27,32,33,35–37,39,41] studies, respectively.

Three studies [30,35,41] on neuroimaging found beneficial results regarding lesion size in the MSCs group using a higher intact volume ratio in the contralesional site and/or ipsilesional hemispheric volume or in general.

Amongst the ten studies using immunohistochemical methods to quantify lesion size, nine [22,23,27,32,33,35,37,39,41] of these studies demonstrated the improvement in pathological changes in animals receiving MSCs treatment compared to the control group (Supplement).

Markers for neurogenesis, apoptosis, neuronal development

Several studies (n=4) reported an increased neurogenesis after application of MSCs [29,31,39,41] (Supplement). This was evident for different time points and brain regions. In the case of a second dose, used in the study of van Velthoven et al. no additional effect was observed. [29] Furthermore, three studies showed that synaptic plasticity was improved after MSCs treatment. [27,29,41] In addition, five studies measured the markers of white matter injury, showing the increase in BrdU/Olig2 cells, decrease in MAP2 and MBP loss, increased MPB optical density, and lateral arborisation in animals with PAIS treated with MSCs. Angiogenesis was evaluated in three studies. Two of these studies [27,39] demonstrated the improvement in blood flow in the peri-infarct region [39] and increased collagen IV, [39] IL-8, and VEGF levels.[22] However, the study by Cho et al. noted the improvement in angiogenesis, measured by FGF-2, VCAM, MMP-2, CD31+ cells, only in animals that received MSCs + enriched environment, while no differences were noted in angiogenesis between PAIS + MSCs and PAIS + Vehicle group animals. [34] Apoptosis was reported by only one study, [35] reporting the decrease in TUNEL-positive cells in the MSCs group of animals.

Markers for inflammation

Markers for inflammation were reported in eight studies. Astrogliosis was assessed in four studies [26,34,35,41] showing the controversial results. One study reported the data on pro-inflammatory cytokines (both mRNA and blood levels of IFN-γ, IL 17, TNFα) showing the decrease in the levels in the animals treated with MSCs, [23] while the other study demonstrated the up-regulation of HO-1 and Nrf2 mRNA and protein expressions. [22] (Supplement)

Distribution of MSCs

Eight studies assessed the distribution of transplanted MSCs. [22,23,25,26,32,35,36,40] (Supplement) All studies reported that MSCs migrated into the ischemic region but with different manifestations and expressions. Concerning the administration route, one study showed that intravenous transplantation significantly recruited more MSCs to the injured brain area than intraperitoneal administration [26] while another study could detect no differences. [40] To date, the distribution of MSCs was assessed at 24h post-administration in the study of Ohshima et al., while Zhang et al. assessed it at 35th day post-transplantation.
